# A European Research Agenda for Geriatric Emergency Medicine: a modified Delphi study

**DOI:** 10.1007/s41999-020-00426-8

**Published:** 2020-11-21

**Authors:** Simon P. Mooijaart, Christian H. Nickel, Simon P. Conroy, Jacinta A. Lucke, Lisa S. van Tol, Mareline Olthof, Laura C. Blomaard, Bianca M. Buurman, Zerrin D. Dundar, Bas de Groot, Beatrice Gasperini, Pieter Heeren, Mehmet A. Karamercan, Rosa McNamara, Aine Mitchell, James D. van Oppen, F. Javier Martin Sanchez, Yvonne Schoon, Katrin Singler, Renan Spode, Sigrun Skúldóttir, Thordis Thorrsteindottir, Marije van der Velde, James Wallace

**Affiliations:** 1 Department of Gerontology and Geriatrics, Leiden University Medical Centre, PO Box 9600, 2300 RC Leiden, The Netherlands; 2 Institute for Evidence-Based Medicine for Older People (IEMO), Leiden, The Netherlands; 3 Department of Emergency Medicine, University Hospital Basel, University of Basel, Basel, Switzerland; 4 Department of Health Sciences, University of Leicester, Leicester, UK; 5 Department of Emergency Medicine, Spaarne Gasthuis, Haarlem, The Netherlands; 6 Section of Geriatric Medicine, Department of Internal Medicine, Amsterdam Public Health Research Institute, Amsterdam UMC, University of Amsterdam, Amsterdam, The Netherlands; 7 Department of Emergency Medicine, Necmettin Erbakan University Meram Faculty of Medicine, Konya, Turkey; 8 Department of Emergency Medicine, Leiden University Medical Centre, Leiden, The Netherlands; 9 Department of Geriatrics and Rehabilitation, Santa Croce Hospital, Azienda Ospedaliera Ospedali Riuniti Marche Nord, Fano, Italy; 10 Department of Public Health and Primary Care, KU Leuven, Leuven, Belgium; 11 Department of Geriatric Medicine, University Hospitals Leuven, Leuven, Belgium; 12 Research Foundation-Flanders (FWO), Brussels, Belgium; 13 Department of Emergency Medicine, Gazi University School of Medicine, Ankara, Turkey; 14 Department of Emergency Medicine, St. Vincent University Hospital, Dublin, Ireland; 15 Department of Emergency Medicine, Sligo University Hospital, Sligo, Ireland; 16 Emergency Department, University Hospitals of Leicester NHS Trust, Leicester, UK; 17 Emergency Department, Hospital Clínico San Carlos, Instituto de Investigación Sanitaria Hospital Clínico San Carlos (IdISSC), Universidad Complutense de Madrid, Madrid, Spain; 18 Department of Emergency Medicine and Department of Geriatrics, Radboud Institute for Health Sciences, Radboud University Medical Center, Nijmegen, The Netherlands; 19 Department of Geriatrics, Klinikum Nürnberg, Paracelsus Private, Medical University, Nuremberg, Germany; 20 Department of Emergency Medicine, Charité University Hospital, Berlin, Germany; 21 Research Institute in Emergency Care, Landspitali National University Hospital of Iceland, Reykjavík, Iceland; 22 The Icelandic Gerontological Research Institute, Reykjavík, Iceland; 23 Department of Geriatrics, Amphia Hospital, Breda, The Netherlands; 24 Emergency Department, Warrington and Halton Hospitals NHS Teaching Trust, Warrington, England, UK

**Keywords:** Geriatric Emergency Medicine, Research prioritisation

## Abstract

**Purpose:**

Geriatric Emergency Medicine (GEM) focuses on delivering optimal care to (sub)acutely ill older people. This involves a multidisciplinary approach throughout the whole healthcare chain. However, the underpinning evidence base is weak and it is unclear which research questions have the highest priority. The aim of this study was to provide an inventory and prioritisation of research questions among GEM professionals throughout Europe.

**Methods:**

A two-stage modified Delphi approach was used. In stage 1, an online survey was administered to various professionals working in GEM both in the Emergency Department (ED) and other healthcare settings throughout Europe to make an inventory of potential research questions. In the processing phase, research questions were screened, categorised, and validated by an expert panel. Subsequently, in stage 2, remaining research questions were ranked based on relevance using a second online survey administered to the same target population, to identify the top 10 prioritised research questions.

**Results:**

In response to the first survey, 145 respondents submitted 233 potential research questions. A total of 61 research questions were included in the second stage, which was completed by 176 respondents. The question with the highest priority was: Is implementation of elements of CGA (comprehensive geriatric assessment), such as screening for frailty and geriatric interventions, effective in improving outcomes for older patients in the ED?

**Conclusion:**

This study presents a top 10 of high-priority research questions for a European Research Agenda for Geriatric Emergency Medicine. The list of research questions may serve as guidance for researchers, policymakers and funding bodies in prioritising future research projects.

## Key Summary Points

**Aim:**

To provide an inventory and prioritisation of research questions amongst GEM professionals throughout Europe.

**Findings:**

A list of 10 research questions was identified and prioritised.

**Message:**

The list of research questions may serve as guidance for scientists, policymakers and funding bodies in prioritising future research projects.

## Introduction

Geriatric Emergency Medicine (GEM) focuses on opportunities to improve outcomes for older people by applying the knowledge and skills required for prevention, diagnosis, and management of urgent care presentations [1, 2]. Older people are already core users of Emergency Medicine (EM) [1, 3]. Providing care for older people is complex, since often there is multimorbidity or frailty and patients may present with non-specific complaints and vital signs which may need to be interpreted differently. Furthermore, GEM is delivered both in the Emergency Department (ED) and in other healthcare settings and by various types of professionals, such as nurses, physiotherapists and physicians, often in a multidisciplinary manner. The knowledge gap caused by lack of scientific evidence in this patient group hinders care professionals in the field of GEM in providing older patients with appropriate diagnostic and therapeutic interventions [4].

Evidence regarding optimal care for the vulnerable older population is lacking, and it is still unclear which research topics have the most added value in the improvement of GEM and which should be prioritised above others [1, 5].

The present study aimed to provide an inventory and prioritisation of research questions among healthcare professionals in Europe regarding the improvement of urgent care for older people.

## Methods

### Study design

The development of a research agenda on GEM at a European level is a joint initiative of the European Society for Emergency Medicine GEM section (EUSEM GEM section) and the European Geriatric Medicine Society GEM Special Interest Group (EuGMS GEM SIG). The Delphi method is an acknowledged consensus method used for determining the extent of agreement on a certain topic [6]. This two-stage modified Delphi method was derived from the PREDICT prioritisation study by Deane et al. [7].

**Fig. 1:**
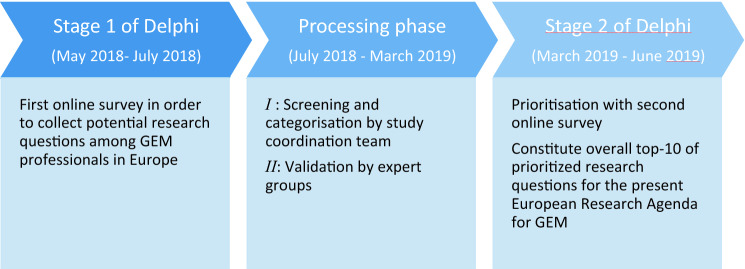
Overview of research process in order to gain insight into the knowledge gap in the field of Geriatric Emergency Medicine by conducting two modified Delphi stages

We used two rounds of surveys: survey 1 was performed during stage 1 and survey 2 was performed during stage 2. In stage 1, the divergent phase, a non-limited list of potential research topics and questions, was administered using an online survey among care professionals throughout Europe following the modified Delphi process. In the processing phase, the convergent phase, the collected research questions and topics were screened, validated, and categorised during expert panel meetings. Subsequently, in stage 2, the remaining research questions were prioritised using a second online survey distributed among care professionals, including the participants of stage 1 (Fig. 1).

#### Expert panel

The expert panel of the processing phase consisted of emergency physicians who are members of the EUSEM GEM section and geriatricians and geriatric nurses who are members of EuGMS GEM SIG.

### Stage 1 of Delphi: collecting potential research questions

From 2 May 2018 to 6 July 2018, the first online survey was published and distributed among all members of EUSEM and EuGMS throughout all European countries by email, using both organisations’ networks to contact national organisations of interest. Particular care was taken to collect data from all European countries by searching the internet for national organisations, with equal representation of both emergency physicians and geriatricians as well as nurses from both fields and representatives of other professions involved in GEM, e.g. physiotherapists, occupational therapists, pharmacists, and dieticians.

Input on potentially relevant research questions was collected by proposing the following question: ‘When reflecting on your clinical practice in the field of Geriatric Emergency Medicine, which questions with the aim of improving the emergency care for acutely ill older people should be addressed in future research?’ Inputs were collected through the website https://www.geriemeurope.eu/research-agenda/ after which stage 1 was closed and the survey was no longer available online. This first stage of collection resulted in a provisional long list of potential research questions.

### Processing phase I: screening and categorisation

*Processing phase I* was devoted to the screening of the long list in order to discard the clearly inappropriate research questions, duplicated research questions or already answered research questions and to categorise the remaining inputs. Groups of questions were assigned by category to couples consisting of two experts (one from geriatrics and one from Emergency Medicine), who independently screened the questions for the following criteria:

1. Is the input relevant to the focus of the survey regarding older people with acute disease in diverse urgent healthcare settings? Clearly, out-of-focus inputs were discarded.
2. Is the input a clear and specific question? Unclear and unspecific inputs were discarded. A question was regarded unclear, for instance, whether the experts were uncertain which determinant, comparison, intervention or outcomes were mentioned, or unspecific if only one of a determinant or outcome was mentioned, for example just a simple word such as ‘atrial fibrillation’.
3. Has the question already been answered by previous or ongoing research? Already answered questions were discarded. This was ascertained by consulting the expert group of authors on the one hand and by performing PubMed searches on the other hand.
4. Is the question a duplication of a previous input? Duplications were excluded.

Questions were either discarded or passed to the next phase. In case of disagreement between the experts, consensus was reached by discussion in the expert panel.

The remaining questions were then categorised into (within) a list of topic areas that was generated in a previous expert panel meeting after reviewing the literature: (1) organisation of care (structural, processes and attitude); (2) screening; (3) triage; (4) evaluation and management; (5) diagnostics; (6) geriatric syndromes in emergency settings; (7) disposition; and (8) ethics. Finally, a categorised shortlist of questions was obtained, which served as input for processing phase II*.* Discarded inputs were saved for later analysis on usability for clinical or educational purposes.

### Processing phase II: validation

The aim of this phase was to validate the research questions of the shortlist obtained by screening in processing phase I and to specify them when necessary. All research questions of the shortlist were transformed into Population, Intervention, Comparison, Outcome (PICO) format, if possible. Subsequently, a literature search was performed for all PICOs by members of the expert panel. Additionally, each question of the shortlist was assessed for validity in a face-to-face expert meeting (May 2018, Basel) based on the following criteria:

1. Is the question relevant for the field of GEM throughout Europe? Questions not focusing upon older people (65 + years) in receipt of care in urgent care settings were excluded.
2. Is there current existing evidence available to answer the question? A question was excluded the expert panel agreed that existing evidence could answer the question.
3. Can the question be feasibly answered in terms of resources (money, time, ethics)?

All expert panel members had to reach consensus about the validity assessment of each question individually. During the validity assessment, inputs were checked for their previous allocated category as well. An additional teleconference was scheduled to discuss the doubtful inputs. Following this teleconference, the eight categories from processing phase II were merged into five categories. After reaching consensus on all inputs’ validity and allocated category, the final list of research questions was composed.

### Stage 2 of Delphi: prioritisation by participants

Using the final list of research questions resulting from processing phase II, a second online survey was conducted among care professionals throughout Europe, including all respondents of stage 1. The survey was set out online from 1 March 2019 until 6 May 2019 on the same website used in stage 1 (see above), and one reminder was sent. The following question was asked: ‘When reflecting on your clinical practice in the field of Geriatric Emergency Medicine, how important are the following questions to you in terms of need for future research?’ Subsequently, respondents were asked to rate each research question of the ‘validated long list’ individually by allocating a percentage, ranging from 0 to 100%, with a slider indicating the importance of the question, 0% indicating not important, and 100% percent indicating very important.

After collecting the allocated scores, the questions were ranked according to the highest average of the ranking percentage. As determined in advance, the ten highest ranking questions constituted an overall top 10 of research questions, and therefore, the consensus regarding the content of the present European Research Agenda for Geriatric Emergency Medicine was reached. Furthermore, two subdivisions consisting of multiple subgroups were made. The first subdivision concerned four GEM professions working in the hospital setting, namely emergency physicians/acute medicine; geriatricians; ED nurses; and geriatric nurses. The second subdivision was made between primary care professionals, secondary care professionals and others. For each subgroup, a top 5 of prioritised research questions was constituted resulting from their submitted ranking scores. The degree of representation of each subgroup in the overall top 10 was determined by analyzing each top 5 separately on overlapping research questions.

## Results

After closing the online survey of stage 1, 233 research questions from 145 respondents throughout Europe were collected (Table 1). In total, ten different professions in the field of GEM were represented in this first survey. The following three professions within the geriatric emergency care chain were represented the most: emergency physician/acute medicine (*n* = 50); geriatrician (*n* = 40); and ED nurse (*n* = 11). On 6 May 2019, the second online survey—belonging to stage 2—was closed. In those four weeks, 176 respondents did fill out the survey and prioritised the research questions of the ‘validated long list’ (Table 1). The same three professions were represented the most in this second survey: geriatrician (*n* = 72); emergency physician/acute medicine (*n* = 65); and ED nurse (*n* = 9). In total, 25 European countries were represented among all respondents.

**Table 1:** Baseline characteristics respondents from the first and second survey

	Stage 1 of Delphi (first survey)	Stage 2 of Delphi (second survey)
No. of respondents	145	176
Professions		
Emergency physician/acute medicine	50 (34.5%)	65 (36.9%)
Geriatrician	40 (27.6%)	72 (40.9%)
General practitioner	9 (6.2%)	2 (1.1%)
Other physician	8 5.5.%)	6 (3.4%)
ED nurse	11 (7.6%)	9 (5.1%)
Geriatric nurse	4 (2.8%)	8 (4.5%)
Other nurse	7 (4.8%)	4 (2.3%)
Physiotherapist	9 (6.2%)	5 (2.8%)
Other healthcare worker	5 (3.4%)	–
Researcher	2 (1.4%)	–
Unknown	–	5 (2.8%)
Country		
Austria	1 (0.7%)	–
Belgium	5 (3.4%)	5 (2.8%)
Bosnia Herzegovina	1 (0.7%)	–
Croatia	1 (0.7%)	1 (0.6%)
Cyprus	1 (0.7%)	–
Czech Republic	1 (0.7%)	1 (0.6%)
Denmark	6 (4.1%)	5 (2.8%)
Finland	3 (2.1%)	2 (1.1%)
France	–	3 (1.7%)
Germany	1 (0.7%)	3 (1.7%)
Iceland	3 (2.1%)	12 (6.8%)
Ireland	2 (1.4%)	13 (7.4%)
Italy	2 (1.4%)	47 (26.7%)
The Netherlands	20 (13.8%)	14 (7.9%)
Norway	–	1 (0.6%)
Poland	1 (0.7%)	1 (0.6%)
Portugal	–	1 (0.6%)
Romania	1 (0.7%)	–
Slovakia	1 (0.7%)	–
Slovenia	1 (0.7%)	9 (5.1%)
Spain	58 (40.0%)	23 (13.1%)
Sweden	–	2 (1.1%)
Switzerland	3 (2.1%)	5 (2.8%)
Turkey	3 (2.1%)	14 (7.9%)
UK	22 (15.2%)	11 (6.2%)
Non-European	6 (4.1%)	2 (1.1%)
Unknown	2 (1.4%)	1 (0.6%)

The first survey, belonging to stage 1 of Delphi, was administered to various professionals working in the field of GEM, with the aim of making an inventory of potential research questions. In stage 2 of Delphi, the remaining research questions—research questions that were collected during stage 1 and that passed the subsequent screening and validation phase—were ranked based on relevance by administering a second survey to the same target population to identify the top 10 of prioritised research questions concerning GEM

**Fig. 2:**
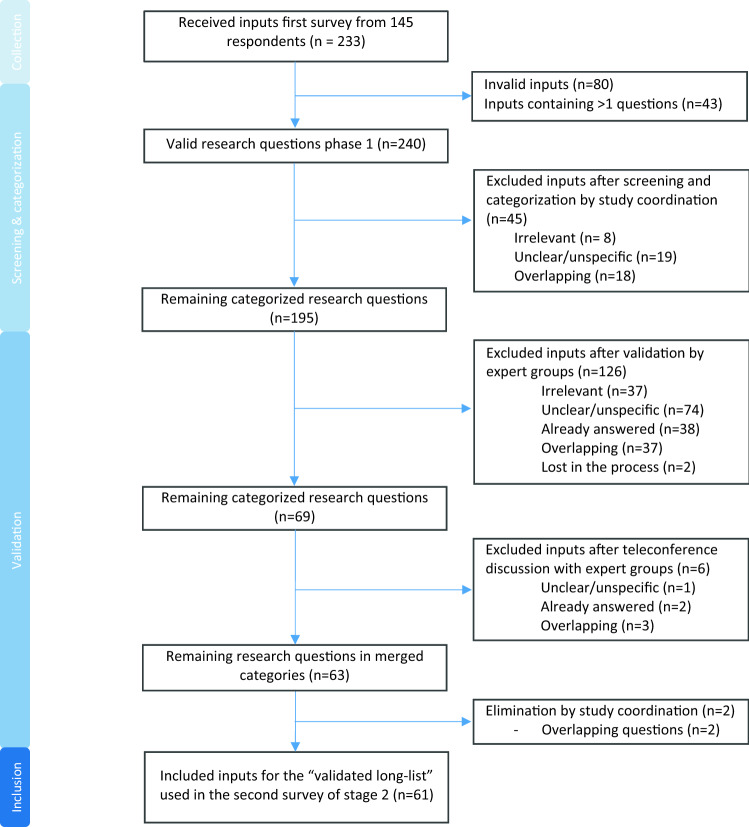
Flowchart representing the screening and categorization process (processing phase I) and the validation (processing phase II) of the received inputs resulting from the first online survey of stage 1

All 233 received inputs resulting from the first online survey were collected and screened for invalid inputs and the presence of multiple questions in one submitted input, resulting in a list of 240 valid research questions (Fig. 2). In the subsequent processing phase I, 45 (18.8%) inputs were excluded based on the following criteria: irrelevance (*n* = 8); unclear (*n* = 8); and the presence of overlapping content (*n* = 18), or a combination of these three (*n* = 11). Of all remaining categorised 195 inputs that passed processing phase I, 126 (52.5%) inputs were excluded after validation in processing phase II by expert groups based on irrelevance (*n* = 37); unclear (*n* = 74); the presence of overlapping content (*n* = 37); and already answer available (*n* = 38). Several inputs were excluded based on more than one criterion. Finally, another eight inputs (3.3%) were excluded following the scheduled teleconference with the expert groups and the final check by the study coordination, resulting in 61 (25.4%) remaining validated inputs, which were implemented in the second survey used in stage 2.

After processing all submitted ranking scores from the second survey and calculating the average scores of all research questions individually, the top 10 comprising the ten research questions with the highest average scores was composed (Table 2). The mean score of all questions was 70.1%. The next three research questions received—with more than 80%—the highest average scores: (1) is implementation of elements of CGA, such as screening for frailty and geriatric interventions, effective in improving outcomes for older patients at the ED? (*M* = 83.5%); (2) which interventions in older ED patients are effective in reducing ED or hospital length of stay? (*M* = 81.0%); and (3) is ‘hospital at home’ effective and cost-effective in improving outcomes in older ED patients? (*M* = 80.6%). The question with the lowest ranking score was: are ED-based vaccination programs effective and cost-effective in decreasing the rate of infectious disease-related ED presentations, hospital admissions and mortality? (*M* = 51.4%).

**Table 2:** Overall top 10 with ranking scores resulting from stage 2

Research questions included in top 10	Ranking score (%)
1. Is implementation of elements of CGA, such as screening for frailty and geriatric interventions, effective in improving outcomes for older patients?	83.5
2. Which interventions in older ED patients are effective in reducing ED or hospital length of stay?	81.0
3. Is ‘hospital at home’ effective and cost-effective in improving outcomes in older ED patients?	80.6
4. Is the presence in the ED of a geriatrician or geriatric nurse effective in improving outcomes for older ED patients?	79.6
5. What interventions are effective in reducing ED visits of older adults?	79.5
6. Does additional geriatric training of ED nurses improve patient outcomes in older ED patients?	79.5
7. Is assessment of frailty effective in reducing the number of unscheduled reattendances of older patients visiting the ED?	78.5
8. Do education and training interventions focusing on geriatric syndromes of ED staff improve outcomes for older patients in the ED?	78.0
9. Which elements of CGA, such as screening for frailty and geriatric interventions, are feasible in the ED?	77.8
10. Which alternative models of care outside the ED are safe and effective to deliver geriatric emergency medicine to older patients who would otherwise come to the ED?	77.4

The ranking scores are calculated from all submitted scores that were allocated to each research question by the respondents of the second survey

*CGA* comprehensive geriatric assessment, *ED* Emergency Department

In addition to the overall top 10, a subdivision was made between emergency physicians/acute medicine (42%), geriatricians (47%), ED nurses (6%) and geriatric nurses (5%) as GEM professionals working in secondary care*.* Table 3 shows the corresponding top 5 per subgroup based on the data of the second survey. The top 5 of emergency physicians/acute medicine is completely represented in the overall top 10*.* Out of the top 5 of both the geriatricians and ED nurses, the first four questions are present in the overall top 10. Finally, out of the top 5 of the geriatric nurses three research questions are represented in the overall top 10 and are the only subgroup in this subdivision that did not prioritise the number one of the overall top 10 in their top 5*.* Furthermore, another subdivision was made based on the respondent distribution among primary care (4%), secondary care (87%) and others (9%). Table 4 presents the top 5 for each subgroup separately. Of the primary care group—consisting of general practitioners and physical therapists—four questions of the top 5 are present in the overall top 10*.* The top 5 of the secondary care group—which consists of emergency physicians/acute medicine, geriatricians, ED nurses, and geriatric nurses—is completely represented in the overall top 10 with (almost) corresponding ranking scores*.* In the group of others—consisting of other physicians, other nurses, and unknown—all excepting the fourth question of the top 5 are notated in the overall top 10.

**Table 3:** Top 5 of research questions for each GEM profession working in secondary care, namely emergency physicians/acute medicine; geriatricians; ED nurses; and geriatric nurses

		Ranking score	Notation in top 10	% in overall list
	Top 5 emergency physicians/acute medicine (*n* = 65)			
1	Is ‘hospital at home’ effective and cost-effective in improving outcomes in older ED patients?	80.3%	3	80.6
2	Is implementation of elements of CGA, such as screening for frailty and geriatric interventions, effective in improving outcomes for older patients?	78.1%	1	83.5
3	Which interventions in older ED patients are effective in reducing ED or hospital length of stay?	78.0%	2	81.0
4	Does additional geriatric training of ED nurses improve patient outcomes older ED patients?	77.7%	6	79.5
5	What interventions are effective in reducing ED visits of older adults?	77.2%	5	79.5
	Top 5 geriatricians (*n* = 72)			
1	Is implementation of elements of CGA, such as screening for frailty and geriatric interventions, effective in improving outcomes for older patients?	86.1%	1	83.5
2	Is the presence in the ED of a geriatrician or geriatric nurse effective in improving outcomes for older ED patients?	85.1%	4	79.6
3	Which interventions in older ED patients are effective in reducing ED or hospital length of stay?	84.1%	2	81.0
4	Which elements of CGA, such as screening for frailty and geriatric interventions, are feasible in the ED?	82.5%	9	77.8
5	Is delivering of elements of CGA in the ED cost-effective?	82.2%	–	75.9
	Top 5 ED nurses (*n* = 9)			
1	Is implementation of elements of CGA, such as screening for frailty and geriatric interventions, effective in improving outcomes for older patients?	91.2%	1	83.5
2	Does additional geriatric training of ED nurses improve patient outcomes older ED patients?	88.7%	6	79.5
3	Is assessment of frailty effective in reducing the number of unscheduled reattendance of older patients visiting the ED?	86.4%	7	78.5
4	Is the presence in the ED of a geriatrician or geriatric nurse effective in improving outcomes for older ED patients?	85.4%	4	79.6
5	Are interventions led by a geriatric nurse effective in improving outcomes for older patients in the ED?	85.2%	–	74.0
	Top 5 geriatric nurses (*n* = 8)			
1	Does additional geriatric training of ED nurses improve patient outcomes older ED patients?	88.9%	6	79.5
2	Is ‘hospital at home’ effective and cost-effective in improving outcomes in older ED patients?	88.7%	3	80.6
3	Are interventions led by a geriatric nurse effective in improving outcomes for older patients in the ED?	87.6%	–	74.0
4	Is assessment of frailty effective in reducing the number of unscheduled reattendance of older patients visiting the ED?	84.4%	7	78.5
5	What support do caregivers of older ED patients experience and what are their needs?	83.5%	–	71.3

The ranking scores are calculated from all submitted scores that were allocated to each research question by the respondents of the second survey

*CGA* comprehensive geriatric assessment, *ED* Emergency Department

**Table 4:** Top 5 of research questions for primary care, secondary care and others

		Ranking score	Notation in top 10	% in overall list
	Top 5 primary care (*n* = 7)			
1	Is implementation of elements of CGA, such as screening for frailty and geriatric interventions, effective in improving outcomes for older patients?	90.3%	1	83.5
2	What interventions are effective in reducing ED visits of older adults?	88.8%	5	79.5
3	What symptoms or signs predict prolonged hospitalisation in older patients?	86.4%	–	68.8
4	Which elements of CGA, such as screening for frailty and geriatric interventions, are feasible in the ED?	85.6%	9	77.8
5	Which interventions in older ED patients are effective in reducing ED or hospital length of stay?	82.6%	2	81.0
	Top 5 secondary care (*n* = 154)			
1	Is implementation of elements of CGA, such as screening for frailty and geriatric interventions, effective in improving outcomes for older patients?	82.3%	1	83.5
2	Which interventions in older ED patients are effective in reducing ED or hospital length of stay?	80.8%	2	81.0
3	Does additional geriatric training of ED nurses improve patient outcomes older ED patients?	80.7%	6	79.5
4	Is ‘hospital at home’ effective and cost-effective in improving outcomes in older ED patients?	80.6%	3	80.6
5	Is the presence in the ED of a geriatrician or geriatric nurse effective in improving outcomes for older ED patients?	80.1%	4	79.6
	Top 5 others (*n* = 15)			
1	Is implementation of elements of CGA, such as screening for frailty and geriatric interventions, effective in improving outcomes for older patients?	92.6%	1	83.5
2	Which elements of CGA, such as screening for frailty and geriatric interventions, are feasible in the ED?	84.0%	9	77.8
3	Is the presence in the ED of a geriatrician or geriatric nurse effective in improving outcomes for older ED patients?	84.0%	4	79.6
4	Are interventions led by a geriatric nurse effective in improving outcomes for older patients in the ED?	82.7%	–	74.0
5	Which interventions in older ED patients are effective in reducing ED or hospital length of stay?	82.3%	2	81.0

The ranking scores are calculated from all submitted scores that were allocated to each research question by the respondents of the second survey

*CGA* comprehensive geriatric assessment, *ED* Emergency Department

## Discussion

After completion of the two stages, a top 10 of high-priority research questions was constituted for the European Research Agenda Emergency Medicine based on the contributions of GEM professionals working throughout Europe. The final prioritised top 10 comprises a diversity of research topics, including diagnostics, preventive interventions, and the capabilities of emergency care professionals.

Considering the wide range of (care) professions in GEM, the chosen study design consisting of two modified Delphi rounds served as a proper method to reach consensus between all parties on the content of this research agenda. By implementing two online surveys, many different potential respondents matching the target population could be reached in a relatively short time span. Additionally, because of online accessibility, the threshold to participate was low. The representativeness would have been higher if more respondents with diverse backgrounds in GEM would have participated in the constitution of the overall top 10. However, despite the differences between the number of respondents per profession in the second survey—e.g. two general practitioners vs. nine ED nurses vs. 72 geriatricians—the results show that the overall top 10 almost completely represents each top 5 of the formulated subgroups (Tables 3, 4). Additionally, the overall top 10 contains a diversity of research topics, which may also indicate a representation of all GEM professionals.

The prioritised research questions very well reflect the knowledge gaps and complexities experienced in the field. For instance, it is still unclear how to best identify older people with frailty in the Emergency Department as screening tools do not perform well [8] and comprehensive geriatric assessment has proven effective [9] but as a whole not to be feasible in the ED. Other approaches, such as the use of readily available data for prediction, may be promising [10, 11], but need further validation, and new approaches, such as the use of machine learning, and implementation science are called for [12]. Another complexity is that delivering Geriatric Emergency Medicine requires a whole system approach and therefore the connection of various professionals. The Acute Frailty Network in the UK is such a network and has shown to result in improvement in patient outcomes [13].

This prioritised list of GEM research topics can serve as research policy for scientists, policymakers, and funding parties in their process of developing research projects and requesting subsidies. In the assessment of the grant proposal, the present research agenda will serve as substantiation for the proposed research topic by emphasizing its importance for the GEM practice. Since evidence and knowledge regarding the provision of optimal care to the vulnerable aged population are lacking, the necessity for future research in the field of GEM is high. Therefore, funding schemes should be allocated to research projects devoted to the prioritised research questions of the present research agenda.

The respondents were different between survey 1 and survey 2. The advantage of this is that the respondents of survey 2 have independently judged the potential research questions on their merits. The disadvantage may be that these second respondents may have missed questions that they have found most relevant or may have misinterpreted the questions.

The first limitation of this study comprises the potential bias resulting from survey fatigue due to the absence of a quasi-randomisation technique in the second survey. The second limitation concerns the representation of all professionals working in GEM throughout Europe. In both surveys, the secondary care professionals are overrepresented compared to primary care professionals. Multiple primary care professionals, e.g. nursing home physicians, district nurses, and occupational therapists, were invited but did not participate in the present study. Additionally, the results showed an unequal representation of different European countries in both surveys, e.g. the overrepresentation of Spain, the UK, and the Netherlands in the first survey (Table 1). The unequal representation of different care professionals and the underrepresentation of several European countries may have influenced the composition of the overall top 10 of research questions. Finally, we did not include older people themselves and their caregivers in the composition of the present research agenda.

This study presents a top 10 of high-priority research questions for a European Research Agenda for Geriatric Emergency Medicine. The list of research questions may serve as guidance for scientists, policymakers and funding bodies in prioritising future research projects.

## Data Availability

Not applicable.
